# Sustained Response to Entrectinib in an Infant With a Germline ALKAL2 Variant and Refractory Metastatic Neuroblastoma With Chromosomal 2p Gain and Anaplastic Lymphoma Kinase and Tropomyosin Receptor Kinase Activation

**DOI:** 10.1200/PO.21.00271

**Published:** 2022-01-27

**Authors:** Diana Treis, Ganesh Umapathy, Susanne Fransson, Jikui Guan, Patricia Mendoza-García, Joachim T. Siaw, Sandra Wessman, Lena Gordon Murkes, Jakob J. E. Stenman, Anna Djos, Lotta H. M. Elfman, John Inge Johnsen, Bengt Hallberg, Ruth H. Palmer, Tommy Martinsson, Per Kogner

**Affiliations:** ^1^ Childhood Cancer Research Unit, Department of Women's and Children's Health, Karolinska Institutet, and Pediatric Oncology, Astrid Lindgren Children's Hospital, Karolinska University Hospital, Stockholm, Sweden; ^2^ Department of Medical Biochemistry and Cell Biology, Institute of Biomedicine, The Sahlgrenska Academy, University of Gothenburg, Gothenburg, Sweden; ^3^ Department of Laboratory Medicine, Institute of Biomedicine, Sahlgrenska Academy, University of Gothenburg, Gothenburg, Sweden; ^4^ Children's Hospital Affiliated to Zhengzhou University, Zhengzhou, China; ^5^ Department of Oncology-Pathology, Karolinska Institutet, Stockholm, Sweden; ^6^ Department of Clinical Pathology, Karolinska University Hospital, Stockholm, Sweden; ^7^ Department of Pediatric Radiology, Astrid Lindgren Children's Hospital, Karolinska University Hospital, Stockholm, Sweden; ^8^ Department of Pediatric Surgery, Astrid Lindgren Children's Hospital, Karolinska University Hospital, Stockholm, Sweden

## Introduction

Despite improved clinical outcomes from multimodal therapy, long-term survival of children with high-risk neuroblastoma (NB) is only about 50%.^1-3^ Targeted approaches may improve these patients' outcomes. The anaplastic lymphoma kinase (ALK) receptor tyrosine kinase (RTK) is one of the few oncogenes identified in NB.^4-8^ *ALK* together with *MYCN* drives NB in a variety of models.^9-12^ ALK is activated by ALKAL ligands,^13-16^ and ALKAL2 overexpression increases onset and penetrance of *Th-MYCN*–driven NB in mice.^10^ *ALKAL2* is located together with *ALK* and *MYCN* on distal 2p, a region often gained in NB^17^ and linked to poor prognosis.^18,19^

Another RTK involved in NB is tropomyosin receptor kinase (TRK)A.^20^ High TRKA expression is a favorable marker,^20^ whereas high expression of the related TRKB is a marker of poor prognosis and progression in NB.^21^ Alternative splicing adds further complexity: a truncated TRKB isoform is preferentially expressed in differentiating NB,^22,23^ whereas an isoform of TRKA that does not bind nerve growth factor is found in unfavorable NB.^24^

Here, we report robust response to RTK inhibition of a patient with NB harboring a rare germline variant in the *ALKAL2* gene with a chromosomal 2p gain and ALK and TRK activity. On the basis of this case, we suggest that NB patients with 2p gain tumors should be investigated for ALK and other RTK signaling activity when possible, even in the absence of genetic mutations, and considered as candidates for targeted therapy.

## Material and Methods

See the Data Supplement.

### Consent.

The patient’s parents have given their written informed consent concerning the submission and publication of this scientific clinical report.

## Results

### Patient presentation.

**FIG 1.**
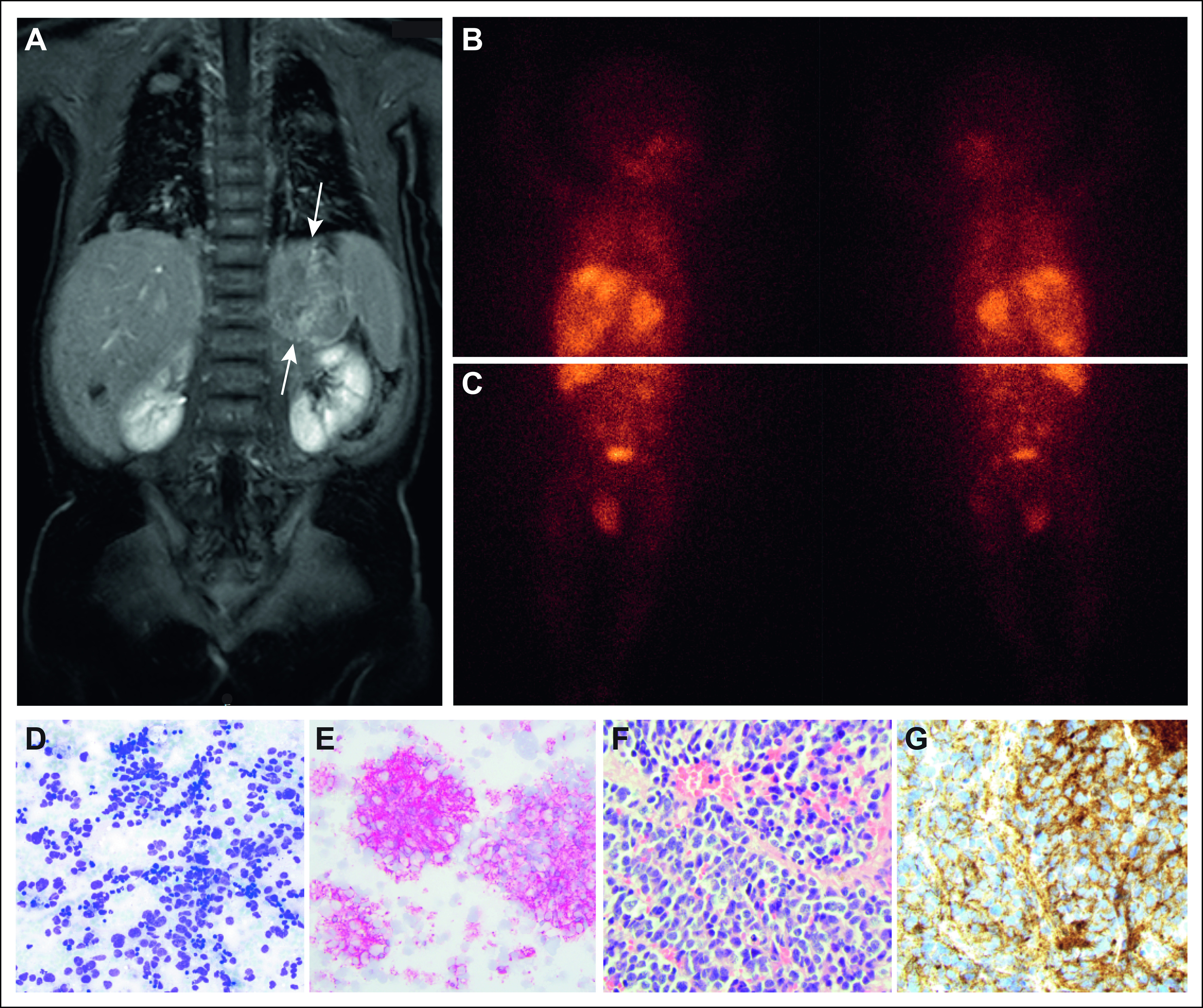
Presentation at diagnosis: (A) T2-weighted MR image showing primary tumor in the left retroperitoneal space and abundant metastases in liver and lungs; (B and C) MIBG scan showing uptake in the primary tumor, retroperitoneal lymph nodes, liver, lungs, femurs, pelvis, and right humerus; (D and E) fine needle aspiration specimens from the primary tumor at 20× enlargement: (D) May-Grünwald-Giemsa staining and (E) synaptophysin staining; (F and G) core needle specimens obtained from the primary tumor at diagnosis at 40× enlargement: (F) hematoxylin-eosin staining and (G) NB84 staining. MIBG, metaiodobenzylguanidine; MR, magnetic resonance; NB, neuroblastoma.

**FIG 2.**
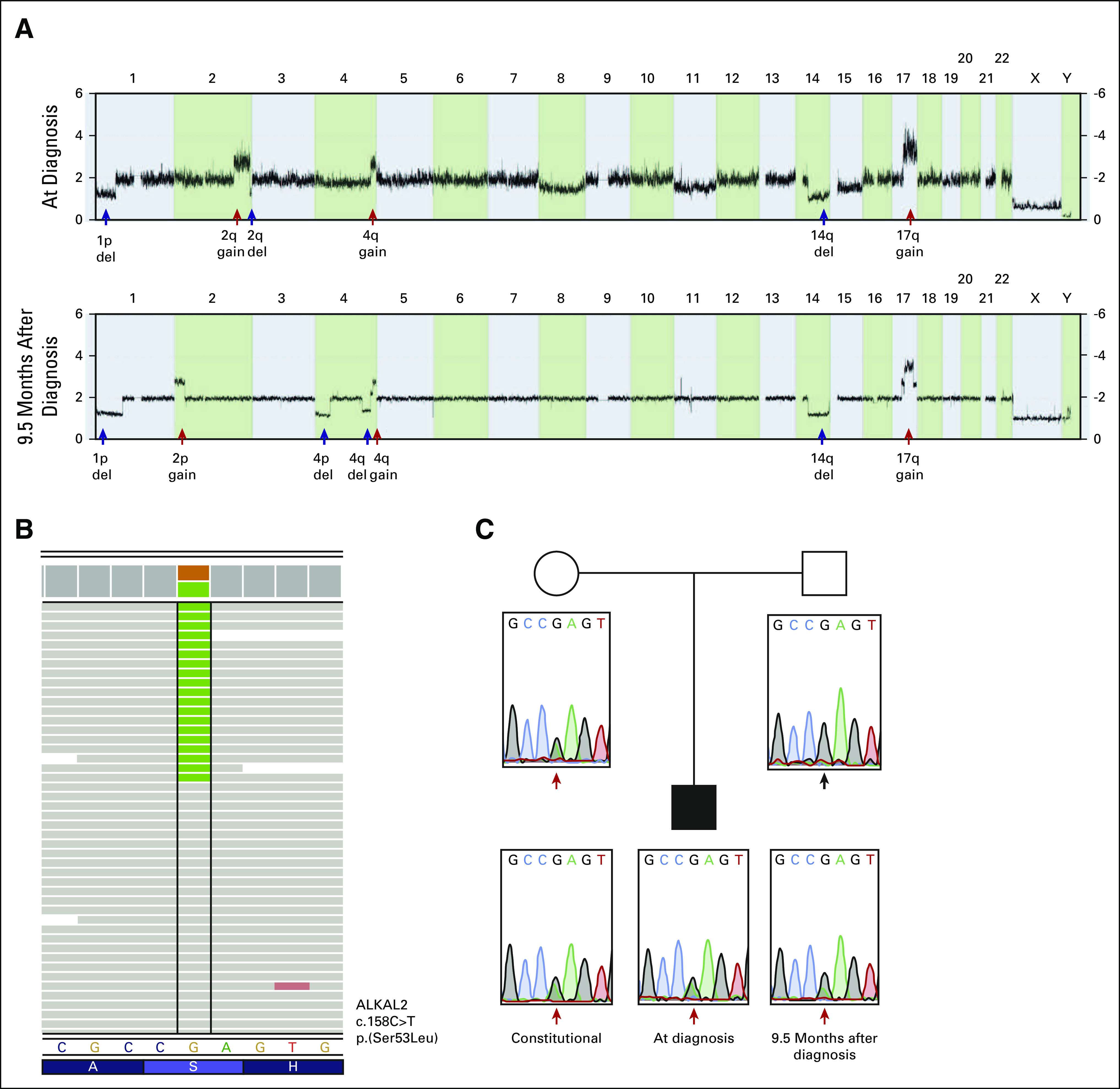
Genomic analysis of tumor samples. (A) Copy number profiling using the Affymetrix HD SNP microarray shows the patient's genomic profiles from tumor biopsy at time of diagnosis (upper panel) and from resected tumor material retrieved 9.5 months after diagnosis (lower panel). Persistent segmental alterations detected in both specimens are deletion of 1p and 14q together with gain of 4q and 17q, whereas additional alterations unique to each sample include 2q gain (at time of diagnosis) and 2p gain together with 4p-del (at time of resection). (B) WGS detected a novel *ALKAL2*^*S53L*^ constitutional variant. (C) Sanger sequencing verified the presence of the *ALKAL2* variant, indicated by red arrows, in the germline of the patient, and subsequent analysis of the patient's parents showed that it was inherited from his healthy mother. WGS, whole-genome sequencing.

A 6-month-old previously healthy boy was referred with a history of vomiting, weight loss, and profuse sweating. Ultrasound showed a tumor at the left adrenal and suspected liver and lymph node metastases. Computed tomography scans confirmed approximately 10 liver and 50 pulmonary metastases but no intracranial metastases. Urine catecholamine metabolites were extremely elevated (Fig 6A). Triple antihypertensive treatment was required to control blood pressure. Further investigations, including magnetic resonance imaging (MRI) and histology, revealed a poorly differentiated unfavorable NB (Figs 1A, 1D-1G). Metaiodobenzylguanidine (MIBG) scan showed uptake in the primary tumor and various metastatic sites (Figs 1B and 1C). Bone marrow involvement was low at 0.1%-0.3%. Single-nucleotide polymorphism (SNP) microarray analysis showed no *MYCN* amplification or 11q deletion but other unfavorable segmental genetic aberrations (Fig 2A, upper panel).

According to the LINES (Low and Intermediate Risk Neuroblastoma European Study) protocol, the patient was allocated to intermediate-risk group 10. The patient showed partial response in MRI and tumor-free bone marrow and partial regression in MIBG after two courses of etoposide and carboplatin (VP/CARBO), but reassessment after courses 3 and 4 showed unaltered tumor volume and persistent metastases. The infant exhibited profuse sweating with persistent elevated blood pressure and catecholamine metabolites. A fourth antihypertensive drug was added and further treatment with CADO (cyclophosphamide, doxorubicin, and vincristine) was scheduled. However, after four courses of CADO, persistent metastases and NB cells in the bone marrow, consultation with the international study lead prompted a therapeutic switch to the high-risk protocol with COJEC induction (cisplatin, vincristine, carboplatin, etoposide, and cyclophosphamide) according to HR-NBL1/SIOPEN.^1^

Restaging after four courses of VP/CARBO, four courses of CADO, and eight courses of COJEC showed NB-free bone marrow. MRI scan showed unaltered findings as compared with two months earlier, and only minor regression compared with initial findings. Stem-cell harvest could not be performed because of insufficient CD34-positive cells.

9.5 months after diagnosis, primary tumor resection with biopsy of para-aortal and mesenteric lymph nodes, kidney, lung and liver was performed. Histopathologic examination revealed viable tumor at six out of eight sites, including MIBG-negative sites. Stem-cell harvest was postponed again because of high bone marrow NB cell involvement. Salvage chemotherapy with topotecan, vincristine, and doxorubicin (TVD)^25^ was initiated with concomitant cyclooxygenase-2 inhibitor celecoxib.^26^ At this point, additional genomic and proteomic studies of resected tumor were initiated, during which time the patient completed four courses of TVD and two ensuing courses of temozolomide and topotecan (TOTEM).^27^

### Genetics of the patient and his tumor.

Genetic assessment by SNP microarray and whole-genome sequencing (WGS) on resected tumor showed several segmental alterations with 2p gain constituting an important change from the genetic profile at diagnosis (Fig 2A). No structural or nonsynonymous variants were detected in genes with established relevance in NB although the 2p breakpoint is located 183 kb distal to *ALK* and fused to *GABRA2* intron 9 at chromosome 4 (Data Supplement). Analysis of constitutional DNA revealed no underlying genetic predisposition for NB, but a novel heterozygous missense variant was detected in *ALKAL2* (NM_001002919.2; c.158C>T, p.(Ser53Leu)), inherited from his healthy mother (Figs 2B and 2C).

### ALKAL2^S53L^ is a functional ALK ligand.

**FIG 3.**
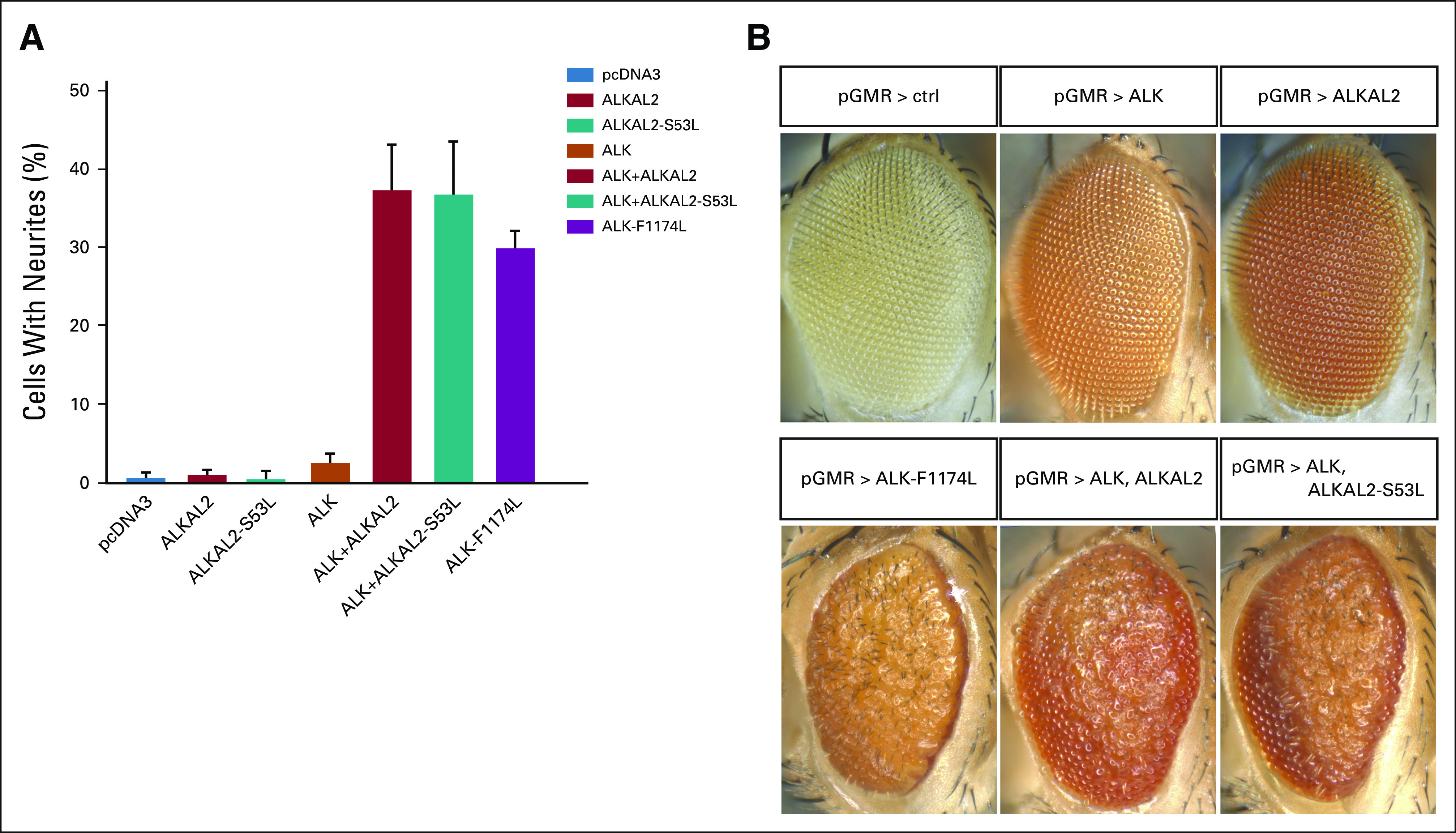
Characterization of the ALKAL2^S53L^ mutant with cell culture and *Drosophila* systems. (A) Neurite outgrowth of PC12 cells as readout for ALK activity was performed with wild-type ALK, ALKAL2, and ALKAL2^S53L^ alone or in combination as indicated. Bars represent the mean percentage ± STD of neurite-carrying cells among GFP-positive cells from three independent experiments. (B) Ectopic expression of human ALK, ALKAL2, and ALKAL2^S53L^ in *Drosophila* eye imaginal discs with the GAL4-UAS system results in a rough eye phenotype that is 100% penetrant upon expression of both the ALKAL2 ligand and the ALK receptor. ALK, anaplastic lymphoma kinase; pGMR, glass multimer reporter vector.

As *ALKAL2* mutations have not been described in NB previously, the ALKAL2^S53L^ variant was evaluated in PC12 cells by neurite outgrowth activity assay.^14,28-30^ Cotransfection of ALKAL2^S53L^ with ALK results in neurite outgrowth at levels comparable with wild-type ALKAL2 (Fig 3A), indicating sustained ability to activate ALK. Further investigation in *D*. *melanogaster*, which offers a clear readout of ALK activation,^14,31^ showed that coexpression of either ALKAL2 or ALKAL2^S53L^ ligands with wild-type ALK resulted in a rough eye (Fig 3B), indicating that both mutated and wild-type ALKAL2 are able to activate human ALK. Taken together, these results indicate that ALKAL2^S53L^ is a functional ligand.

### Protein analysis of tumor material.

**FIG 4.**
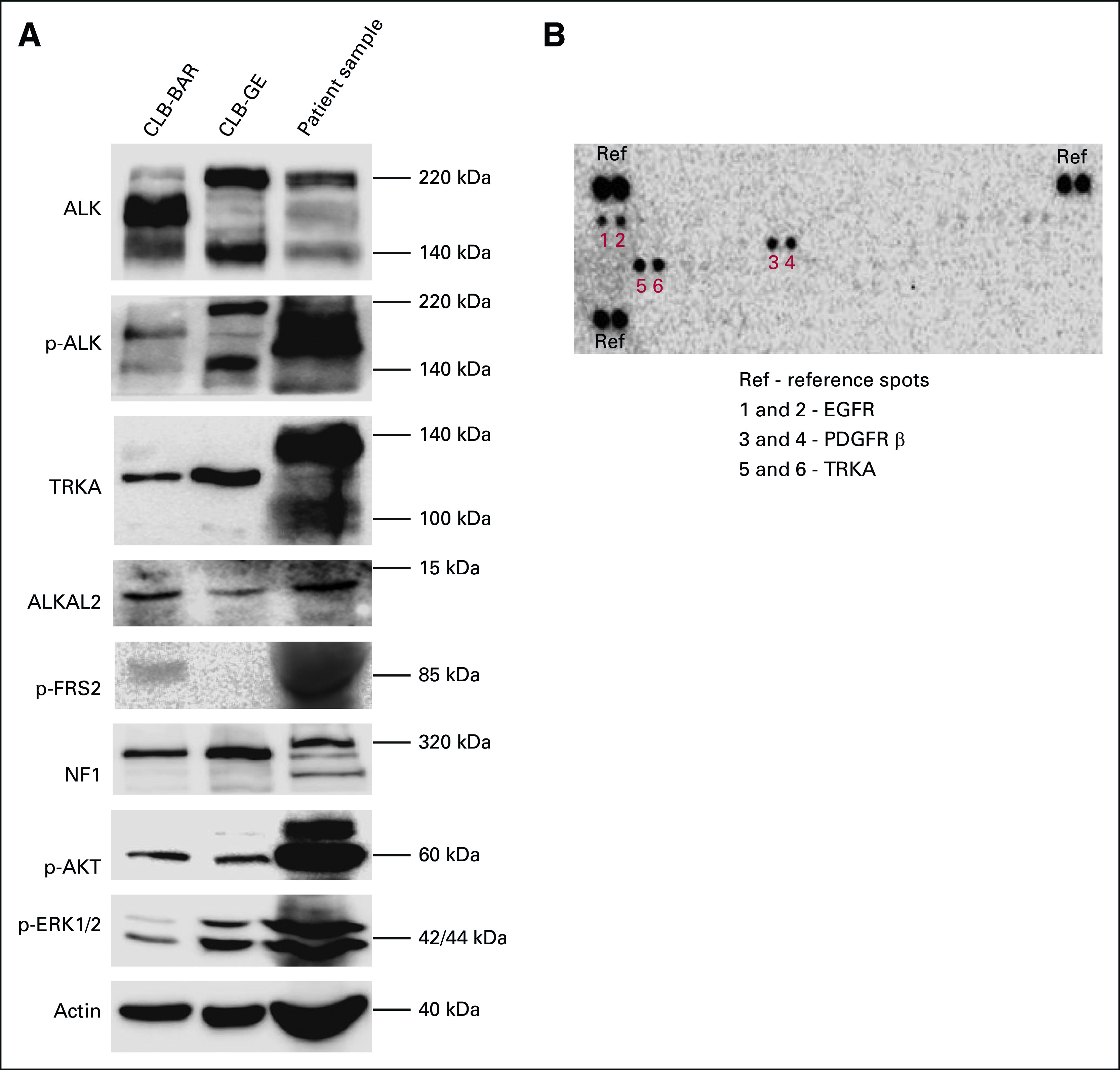
ALK and TRKA are active in the patient tumor sample. (A) Immunoblotting for the indicated proteins in lysates from the ALK-driven NB cell lines CLB-BAR and CLB-GE (used as reference) and patient tumor lysate. (B) Phospho-RTK array analyses of 49 distinct RTKs after incubation with patient tumor lysate. ALK, anaplastic lymphoma kinase; EGFR, epidermal growth factor receptor; NB, neuroblastoma; PDGFR, platelet-derived growth factor receptor; RTK, receptor tyrosine kinase; TRK, tropomyosin receptor kinase.

Immunoblotting of resected primary tumor verified ALK activation, ALKAL2 expression, and phosphorylation of downstream targets: ERK, AKT, and FRS2 (Fig 4A).^32-34^ RTK array analysis identified several additional activated RTKs in the tumor sample (Fig 4B). Specifically, active epidermal growth factor receptor, platelet-derived growth factor receptor beta, and TRKA were detected. High TRKA levels were observed, and a differing molecular weight in comparison with two NB cell lines was noted (Fig 4A). Careful reanalysis of WGS did not identify any genetic aberrations affecting these RTKs or corresponding ligands. The analyses indicated both TRKA and ALK activation. Of the ALK tyrosine kinase inhibitors available, only entrectinib targeted both ALK and TRKA.

### Entrectinib efficiently abrogates ALK activity in NB cell lines.

**FIG 5.**
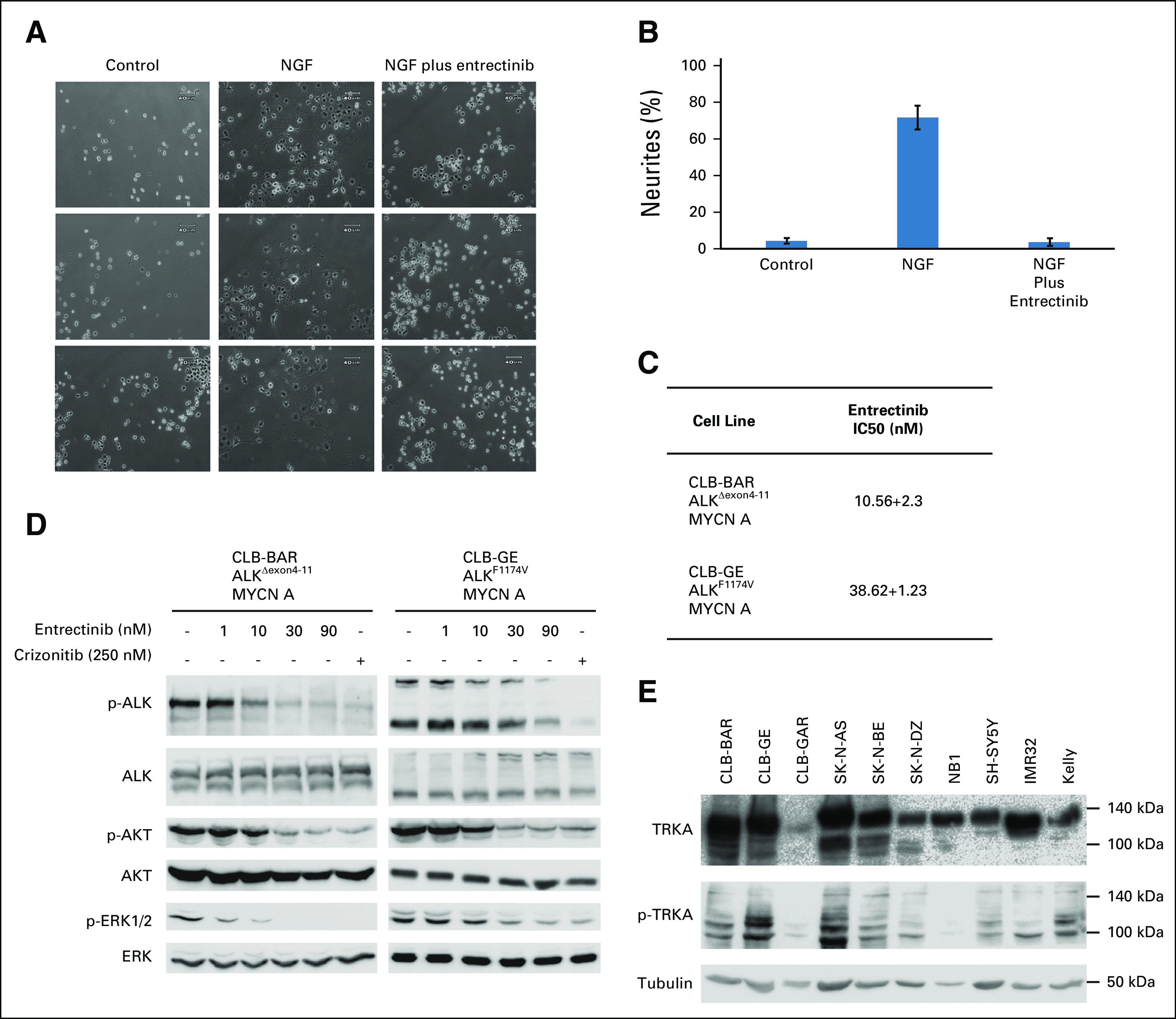
Inhibition of ALK and TRKA by entrectinib. (A and B) PC12 cells were stimulated with NGF (100 ng/mL) alone or in combination with entrectinib. (B) Percentage of neurites was calculated as indicated. (C) IC50 values calculated for viability of NB cell lines treated with increasing concentrations of entrectinib. (D) Inhibition of ALK-driven NB cell lines using crizotinib and entrectinib. ALK-driven CLB-BAR and CLB-GE NB cells were treated for 6 h with either crizotinib or entrectinib as indicated. Cell lysates were analyzed on SDS-PAGE followed by immunoblotting for ALK, phospho-ALK-Y1278, phospho-AKT, AKT, phospho-ERK1 and 2, and pan-ERK. (E) NB cell lysates were analyzed on SDS-PAGE followed by immunoblotting for TRKA, phospho-TRKA, and tubulin as loading control. ALK, anaplastic lymphoma kinase; NB, neuroblastoma; NGF, nerve growth factor; TRK, tropomyosin receptor kinase.

Entrectinib inhibits phosphorylation of TRKB upon brain-derived neurotrophic factor stimulation in SH-SY5Y NB xenografts^35^ and inhibits ALK signaling in NB cells.^36^ Nerve growth factor stimulation of TRKA in PC12 cells caused neurite outgrowth,^37^ which is abrogated by entrectinib (Figs 5A and 5B), supporting a robust TRKA inhibition.^35^ To investigate therapeutic efficacy of entrectinib in NB cells, ALK-driven CLB-BAR and CLB-GE cells were used. A dose-dependent decrease in cell viability was observed with IC50s of 10.6 nM and 38.6 nM for CLB-BAR and CLB-GE, respectively (Fig 5C). In agreement, decreased ALK phosphorylation and inhibition of downstream targets were observed (Fig 5D). Thus, in preclinical experiments, entrectinib inhibits activity of both ALK and TRKA, the latter of which is expressed in most NB cell lines (Fig 5E). Given 2p gain, robust ALK and TRK signaling, and ALKAL2 expression, together with the patient's lack of response to standard treatment, entrectinib therapy was started.

### Patient response to entrectinib.

Because the patient was too young for the ongoing entrectinib RXDX-101-03 trial (inclusion age 2-22 years, NCT02650401), compassionate use was granted by the study sponsor, Ignyta Inc. Treatment with ALK-TRK-ROS1 inhibitor entrectinib started at an oral dose of 200 mg/day (393 mg/m^2^) once daily, increasing to 300 mg (475 mg/m^2^) and 400 mg (540 mg/m^2^) once daily after 10 and 29 months, respectively.

Entrectinib was well tolerated, and no overt acute or long-term toxicity was observed. After 3 months, a solitary episode of syncope with 5-10 seconds of unresponsiveness prompted evaluation with EEG, which turned out normal. From age 2.5 to 3 years, the child developed pathologic fractures occurring sequentially in both tibiae and the right fibula. Suspected new metastases were disproven, and fractures deemed to be caused by previous chemotherapy. One fracture necessitated surgical intervention but all healed under continued entrectinib and celecoxib therapy. No further adverse events or hospitalizations related to medication have been recorded over 4 years of treatment.

**FIG 6.**
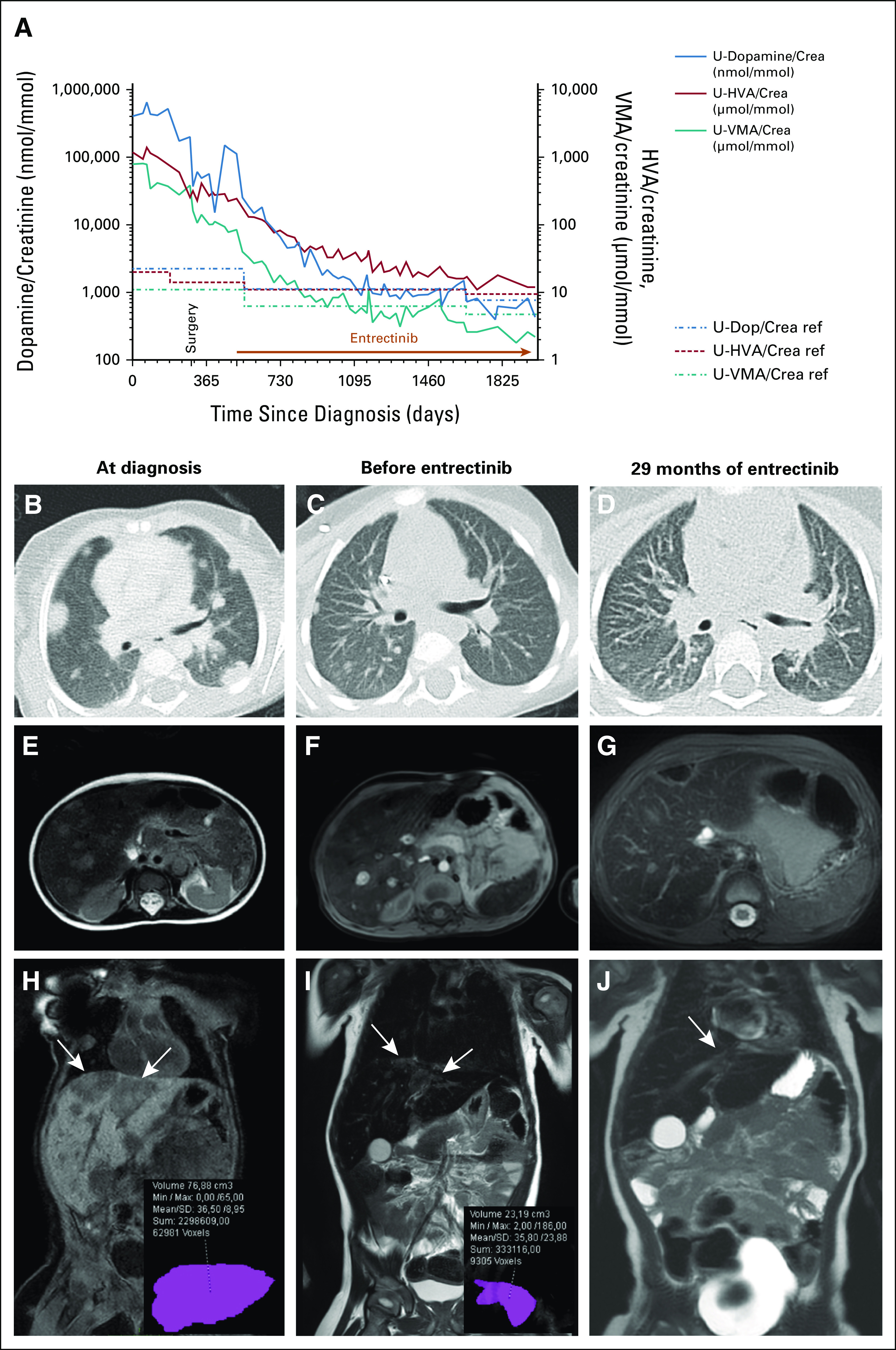
Response to treatment. (A) Urine catecholamine metabolites, logarithmic scale. Catecholamine metabolites (dopamine in blue, HVA in red, and VMA in teal) as NB markers in the patient's urine (molar concentrations/creatinine concentration). Dashed lines represent age-specific reference values. At start of entrectinib treatment, the urine dopamine level was still elevated 50-fold beyond the reference level. CT scans of the lungs showing (B) extensive metastasis at diagnosis, (C) decreasing size and number of metastases after multiple courses of chemotherapy, and (D) further involution after 29 months of entrectinib therapy. Abdominal MR images showing liver metastases (E and H) at diagnosis, (F and I) before start, and (G and J) after 29 months of entrectinib treatment. The metastasis indicated by arrows has been chosen for volumetric analysis [inserts] yielding 76 mL (H) at diagnosis, (I) 23 mL before start of entrectinib, and (J) 0 mL (unmeasurable) after 29 months of entrectinib treatment. MR image acquisition settings are as follows: (E) T2, (F) T1 + gd, (G) T1 + gd (GRE) dixon, (H) T1, (I) T2, and (J) T2. CT, computed tomography; HVA, homovanillic acid; MR, magnetic resonance; NB, neuroblastoma; VMA, vanillylmandelic acid.

Two months after entrectinib treatment initiation, assessment of a liver metastasis showed remaining viable, poorly differentiated cells (Data Supplement) despite generally improved patient condition. However, urine catecholamine metabolites exhibited a gradual decrease to near-normal values (Fig 6A), with repeated computed tomography and MRI scans showing lung and liver metastases decreased to detectability limits (Figs 6B-6J). After 48 months of treatment, radiologic studies demonstrated further resolution of lung and liver metastases (not shown). Bone marrow remained tumor-free at repeated examinations.

The child is alive and well after more than 4 years of continuous and still ongoing therapy with entrectinib and celecoxib. Excluding chemotherapy-induced hearing loss, the patient's social life, development, and growth are completely normal 5.5 years from diagnosis, with catchup in both height and weight during entrectinib, after growth impairment during chemotherapy (Data Supplement).

## Discussion

See the Data Supplement.

In conclusion, we report a patient with refractory metastatic NB who, in the setting of exhausted therapeutic options, responded favorably to entrectinib and reached a stable clinical situation. Although it is unclear whether the mechanism of entrectinib action is mainly via ALK or TRK, this patient represents the first reported case of a 2p gain *ALKAL2* ligand variant, which potentially drives ALK pathway activation in NB. Although the significance of ALK ligand mutation requires further investigation, we suggest that children with NB lacking *ALK* mutation, but with ALK activation and/or mutations in ALK ligands, are considered for ALK-inhibiting targeted therapy.
